# The Origin of Glucocorticoid Hormone Oscillations

**DOI:** 10.1371/journal.pbio.1001341

**Published:** 2012-06-05

**Authors:** Jamie J. Walker, Francesca Spiga, Eleanor Waite, Zidong Zhao, Yvonne Kershaw, John R. Terry, Stafford L. Lightman

**Affiliations:** 1 Henry Wellcome Laboratories for Integrative Neuroscience and Endocrinology, School of Clinical Sciences, University of Bristol, Bristol, United Kingdom; 2 Bristol Centre for Applied Nonlinear Mathematics, Department of Engineering Mathematics, University of Bristol, Bristol, United Kingdom; 3 College of Engineering, Mathematics and Physical Sciences, University of Exeter, Exeter, United Kingdom; University of Cambridge, United Kingdom

## Abstract

Oscillating levels of adrenal glucocorticoid hormones are essential for optimal gene expression, and for maintaining physiological and behavioural responsiveness to stress. The biological basis for these oscillations is not known, but a neuronal “pulse generator” within the hypothalamus has remained a popular hypothesis. We demonstrate that pulsatile hypothalamic activity is not required for generating ultradian glucocorticoid oscillations. We show that a constant level of corticotrophin-releasing hormone (CRH) can activate a dynamic pituitary-adrenal peripheral network to produce ultradian adrenocorticotrophic hormone and glucocorticoid oscillations with a physiological frequency. This oscillatory response to CRH is dose dependent and becomes disrupted for higher levels of CRH. These data suggest that glucocorticoid oscillations result from a sub-hypothalamic pituitary-adrenal system, which functions as a deterministic peripheral hormone oscillator with a characteristic ultradian frequency. This constitutes a novel mechanism by which the level, rather than the pattern, of CRH determines the dynamics of glucocorticoid hormone secretion.

## Abstract 2

Characterization of a peripheral hormonal system identifies the origin and mechanisms of regulation of glucocorticoid hormone oscillations in rats.

## Author Summary

Glucocorticoid steroid hormones, such as cortisol and corticosterone, provide a rapid response to both physical and psychological stress, and act on areas of the brain that influence learning, memory, and behaviour. Glucocorticoids are released from the adrenal glands in near-hourly pulses, which results in oscillating glucocorticoid levels in the blood and in target organs. These hormone oscillations can become disrupted during ageing and in stress-related disease (e.g., major depression), so it is important to identify the underlying mechanisms that govern their dynamics. Although the origin of the oscillations is not known, it is assumed that they are generated by a neuronal “pulse generator” within the brain. In this study, we present data that challenge this hypothesis. We characterize a peripheral hormonal system and show that constant levels of corticotrophin-releasing hormone can induce and regulate hormone oscillations independent of the brain. We also describe mechanisms that can disrupt these oscillations. These findings have important implications for our understanding of glucocorticoid signalling in both health and disease, and will be important for the design of novel treatment strategies that take into account timing of hormone administration to patients undergoing steroid therapy for inflammatory or malignant disease.

## Introduction

A fundamental requisite for survival is the ability to respond and adapt to a changing environment. This ability to respond to change or “stress” becomes more complex in multicellular organisms, and mammals have developed a well-integrated organization of hormonal, neural, and immunological systems that protect them from internal and external threats to their homeostatic state [1]–[3]. One of the most important of these systems, and one that is critical for life, is the hypothalamic-pituitary-adrenal (HPA) axis. This neuroendocrine system regulates the secretion of vital adrenal glucocorticoid hormones (cortisol in humans and corticosterone in rodents), which have major effects on brain and metabolic function and are essential for successful recovery and adaptation to stress [4],[5].

**Figure 1:**
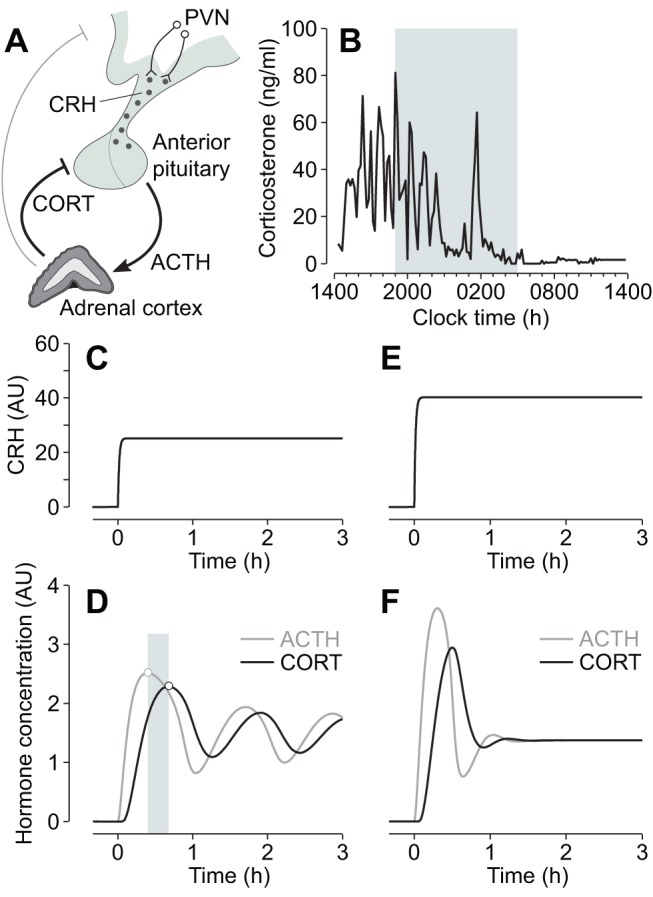
Regulation of glucocorticoid hormone secretion. (A) Negative feedback in the HPA axis plays a key role in regulating glucocorticoid (CORT) secretion. Neurons of the hypothalamic paraventricular nucleus (PVN) secrete CRH into the portal vein, which acts on the anterior pituitary to release ACTH into the general circulation. ACTH activates cells in the adrenal cortex to synthesize and secrete CORT, which in turn feeds back directly on the anterior pituitary to inhibit ACTH secretion, as well as acting at higher centres in the brain, including the hypothalamus and hippocampus. (B) Endogenous corticosterone (the main glucocorticoid in rodents) oscillations in a freely behaving male Sprague-Dawley rat. Shaded region indicates the dark phase. (C–F) Mathematical modelling predicts that ultradian ACTH and glucocorticoid (CORT) oscillations are regulated by a systems-level negative feedback mechanism in the pituitary-adrenal network, independent of pulsatile hypothalamic activity. Numerical simulations show that the pituitary-adrenal network can oscillate under conditions of constant CRH drive to the pituitary (C–D). Oscillations in ACTH and CORT are characterized by a small phase shift (shaded region indicates phase difference between oscillation peaks). For higher levels of CRH drive, the oscillations are rapidly damped to steady-state levels of hormone (E–F). AU, arbitrary units.

Central regulation of glucocorticoid secretion is predominantly coordinated by the hypothalamic peptide corticotrophin-releasing hormone (CRH) [6],[7], the efficacy of which can be significantly potentiated by other hypothalamic factors, most notably vasopressin [8]. CRH is synthesized by parvocellular neurons in the paraventricular nucleus (PVN) of the hypothalamus [9], and secreted into the hypothalamic-pituitary portal circulation from axons terminating in the external zone of the median eminence [10]. Activation of CRH receptors in corticotroph cells of the anterior pituitary results in adrenocorticotrophic hormone (ACTH) secretion into the general circulation, which in turn stimulates glucocorticoid-secreting cells within the adrenal cortex (Figure 1A). Circulating glucocorticoids regulate gene expression in cells throughout the organism via activation of two widely expressed transcription factors—the glucocorticoid receptor (GR) and mineralocorticoid receptor (MR) [11]—as well as acting directly at the cell membrane to initiate more rapid non-genomic signalling processes [12]–[15]. Glucocorticoids also feed back on their own regulatory system to inhibit HPA activity [16],[17]. Inhibition occurs at the level of the hippocampus and hypothalamus, and with particular sensitivity at the level of the anterior pituitary (Figure 1A).

In common with other neuroendocrine systems that signal through pituitary hormone secretion, the HPA axis is characterized by a dynamic ultradian rhythm, which is manifested by oscillating levels of ACTH [18] and glucocorticoid hormones (Figure 1B) both in the blood and in the brain [19]. At the cellular level, glucocorticoid oscillations induce “gene pulsing” through rapid, transient binding of glucocorticoid receptors to promoter elements of glucocorticoid-responsive genes [20],[21]. This dynamic and versatile transcriptional system enables cells to rapidly detect and respond to changes in circulating glucocorticoid levels and provides a sensitive mechanism for the maintenance of homeostasis [22]. Indeed, when the glucocorticoid rhythm is pharmacologically replaced by constant levels of steroid, this results in abnormal gene expression [21], and a desensitization of physiological and behavioural responses to stress [23],[24].

The origin of glucocorticoid oscillations is not known. Since pulsatile patterns of CRH have been detected in cultured hypothalamic explants, median eminence, and portal blood [25]–[27], this has led to speculation that oscillations in the pituitary-adrenal system are a consequence of a neuronal “pulse generator” within the hypothalamus. However, there is a lack of concordance between pulsatile patterns of hypothalamic factors and the ultradian ACTH and glucocorticoid oscillation. In the rat, for instance, CRH pulse frequency [26] is much higher (∼3 pulses/h) than the near-hourly oscillation in ACTH [18] and glucocorticoids (Figure 1B). This suggests that episodic secretion of hypothalamic hormones is not the primary controlling factor of the ultradian rhythm and implies that oscillatory mechanisms exist at a sub-hypothalamic level. This concept of a “peripheral oscillator” is in keeping with in vivo lesion studies demonstrating maintenance of ultradian pulsatility following hypothalamic-pituitary disconnection in the sheep [28], and a loss of circadian but not ultradian glucocorticoid oscillation following suprachiasmatic nucleus lesions in the rat (unpublished data).

Since glucocorticoids rapidly inhibit CRH-induced ACTH secretion from the anterior pituitary [29]–[32], we postulated that this fast inhibitory feedback process provides a potential mechanism within the pituitary-adrenal system for generating oscillatory dynamics. To explore this hypothesis further, and to determine qualitatively the dynamics that result from hormonal interactions between the anterior pituitary and adrenal cortex, we previously developed a mathematical model based on differential equations that incorporates rapid glucocorticoid inhibition of ACTH secretion [33]. Numerical analysis of the model suggests that the pituitary-adrenal system can support self-sustained ACTH and glucocorticoid oscillations with a physiological ultradian frequency, even under conditions of constant CRH drive to the anterior pituitary (Figure 1C–1D). In this model, the ACTH and glucocorticoid oscillations have the same frequency, but they are not synchronous—there is a small phase difference, with ACTH oscillations preceding glucocorticoid oscillations (Figure 1D). The model also predicts that the capacity for this oscillatory response depends on the degree of hypothalamic drive, with higher levels of CRH resulting in damped oscillations to steady-state (i.e., constant) levels of hormone (Figure 1E–1F).

Here we test these modelling predictions in vivo. Our data show that a constant level of CRH can activate the pituitary-adrenal system to produce ultradian hormone oscillations with a physiological frequency, and that this oscillatory activity is critically dependent on the level of hypothalamic drive, with higher levels of CRH resulting in a loss of oscillation. These results demonstrate that pulsatile secretion of hypothalamic CRH is not required for ultradian oscillatory activity in the pituitary-adrenal system, and support our theoretical hypothesis that rapid glucocorticoid inhibition at the level of the anterior pituitary is the primary factor regulating the ultradian dynamics of the system.

## Results and Discussion

To test our hypothesis that the sub-hypothalamic pituitary-adrenal system functions as an ultradian hormone oscillator, we investigated the dynamics of glucocorticoid responses to different levels of constant CRH stimulation in conscious freely behaving male rats. Experiments were performed during the nadir of the circadian cycle (0700–1300 h), when there is minimal endogenous CRH [34],[35], and no pulsatile secretion of corticosterone, the main glucocorticoid in rodents (Figure 1B). Animals were infused intravenously at a constant (i.e., non-pulsatile) rate for this 6-h period with concentrations of CRH in the range 0–2.5 µg/h. Circulating levels of corticosterone were measured in blood samples collected prior to and throughout the infusion using an automated blood-sampling system (see Materials and Methods).

**Figure 2:**
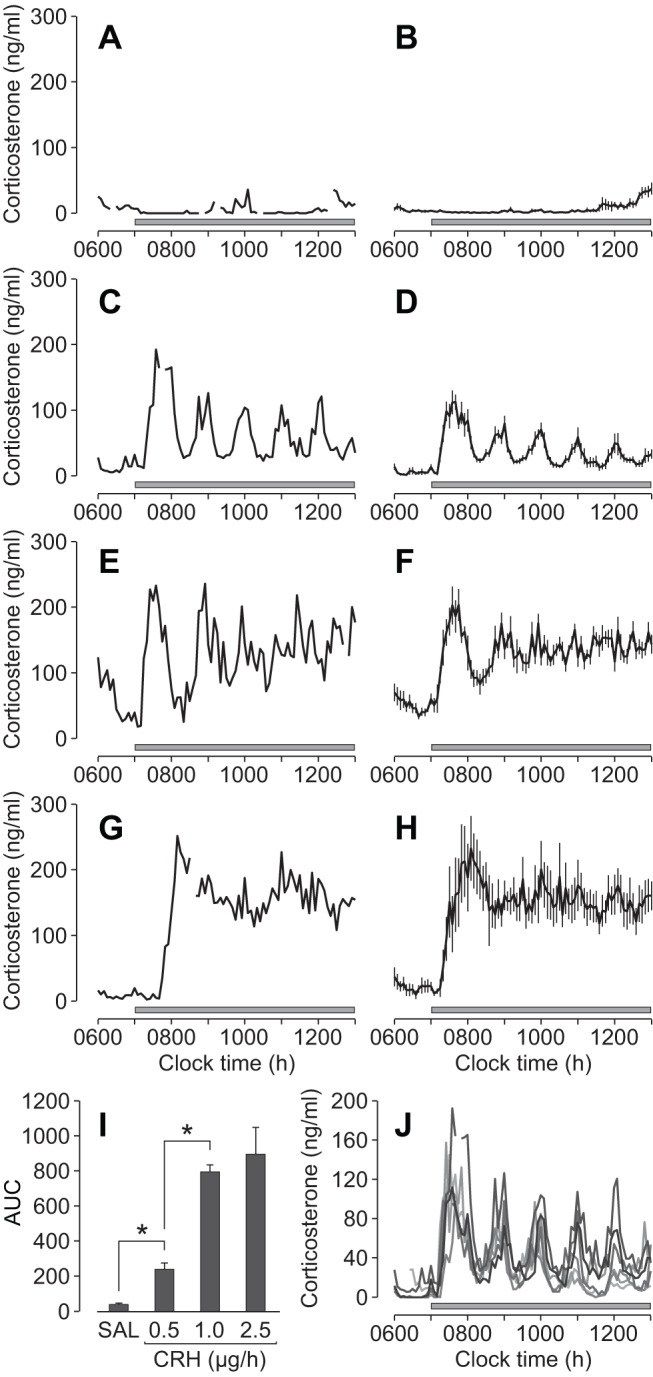
Glucocorticoid response to constant CRH infusion. (A–H) Individual (A, C, E, G) and mean (B, D, F, H) corticosterone responses to constant saline (A–B) or CRH infusion (0.5 µg/h, C–D; 1.0 µg/h, E–F; 2.5 µg/h, G–H). Grey bar indicates the period of infusion; error bars represent mean ± standard error of the mean (SEM) (*n* = 6–8 per group). (I) Dose-dependent effect of CRH on the corticosterone response. Overall effect of the CRH infusion was significant (AUC, *p*<0.0001; Kruskal-Wallis ANOVA on ranks). Error bars represent mean ± SEM (*n* = 6–8 per group); *p<0.001. (J) Synchronous corticosterone oscillations (in individual rats) in response to constant CRH infusion (0.5 µg/h; *n* = 6). Grey bar indicates the period of infusion.

To check that the animals were in a physiological basal state throughout the procedure, we measured corticosterone levels in a group of control animals infused with saline (Figure 2A–2B). Corticosterone levels remained low throughout the duration of the saline infusion and were not significantly different from corticosterone levels measured during the same time period (0700–1300 h) in a group of untreated animals (data not shown), as assessed by analysis of the area under the curve (AUC, *p*>0.38).

In response to CRH infusion, corticosterone levels rose rapidly (Figure 2C–2H) and the overall effect was dose dependent (AUC, *p*<0.0001) (Figure 2I). There was a significant difference between the groups treated with saline and 0.5 µg/h CRH (*p*<0.001), and between the groups treated with 0.5 and 1.0 µg/h CRH (*p*<0.001), but there was no significant difference between the groups treated with 1.0 and 2.5 µg/h CRH (*p*>0.41). This suggests that both of these higher levels of CRH result in maximal pituitary-adrenal activation, which implies a systems-level “ceiling effect” in the pituitary-adrenal network.

Computational modelling suggests that the dynamic activity of the pituitary-adrenal system is fundamentally dependent on the level of hypothalamic stimulation [33]. In agreement with this prediction, constant infusion of CRH at different doses gave rise to different temporal patterns of corticosterone secretion. In line with the modelling hypothesis, constant infusion of CRH (0.5 µg/h) induced ultradian corticosterone oscillations that persisted throughout the infusion period (Figure 2C–2D). In contrast, and also consistent with the qualitative predictions of the model, higher doses of CRH (1.0 and 2.5 µg/h) caused a rapid activation of the adrenals, but the oscillatory component of the response was damped to constant, elevated levels of steroid (Figure 2E–2F, CRH 1.0 µg/h; Figure 2G–2H, CRH 2.5 µg/h).

Although CRH is the predominant ACTH secretagogue in humans and the rat [36], its ability to stimulate ACTH secretion can be potentiated by other hypothalamic neuropeptides, most notably vasopressin [8]. However, the consistency between animals in the timing of the initial corticosterone response to CRH, and the subsequent synchrony in oscillation throughout the infusion (Figure 2J) indicates that the corticosterone response is not dependent on the release of any other endogenous hypothalamic factors, including vasopressin. This is in keeping with previous observations that blocking vasopressin actions on the anterior pituitary (using a vasopressin V1b receptor antagonist) has no effect on endogenous corticosterone oscillations during the circadian peak [37], suggesting that vasopressin is not a significant factor in regulating the ultradian dynamics of the system.

**Figure 3:**
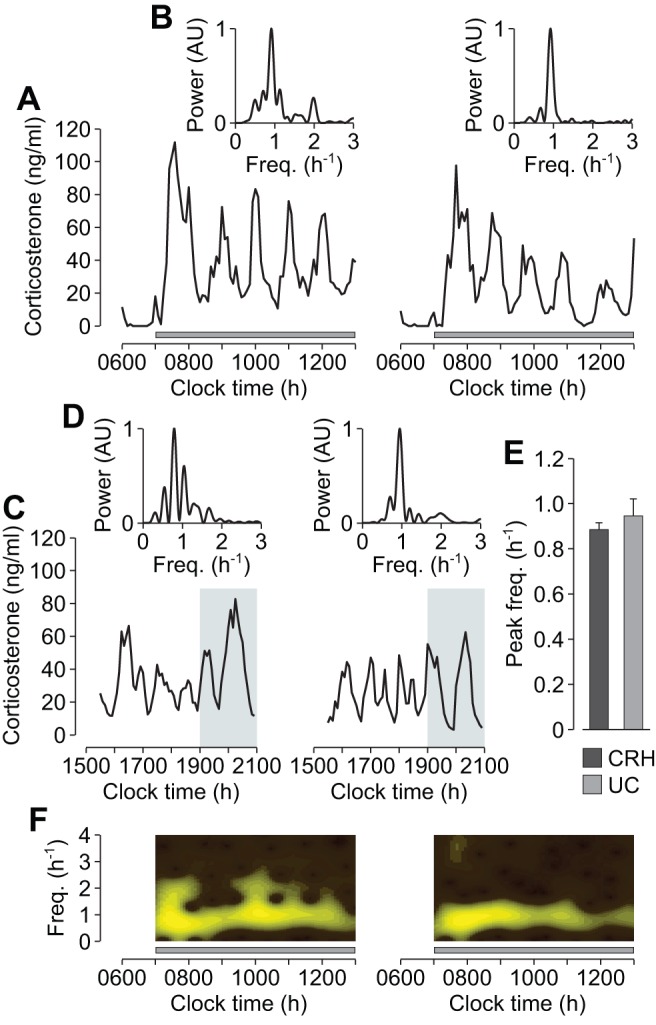
Frequency comparison of CRH-induced and endogenous glucocorticoid oscillations. (A) Individual corticosterone oscillations in response to constant CRH infusion (0.5 µg/h). Grey bar indicates the period of infusion. (B) Normalized power spectra of the corticosterone oscillations in (A). (C) Corticosterone oscillations during the circadian peak in untreated control (UC) rats. Shaded region indicates the dark phase. (D) Normalized power spectra of the corticosterone oscillations in (C). (E) Mean peak frequency (i.e., frequency corresponding to the maximum power in the spectrum) of corticosterone oscillations in response to constant CRH infusion (0.5 µg/h; CRH; *n* = 6), and of corticosterone oscillations during the circadian peak in untreated control rats (UC; *n* = 13). Error bars represent mean ± standard error of the mean (SEM). (F) Frequency evolution of the corticosterone oscillations in (A). AU, arbitrary units.

If the mechanism regulating endogenous corticosterone oscillations during the circadian peak is the same mechanism that is activated by the constant infusion of exogenous CRH, there should be good agreement between the characteristic frequencies of endogenous and CRH-induced oscillations. To test this, we computed the dominant frequency component in the CRH-induced oscillations, and compared this with the dominant frequency component in endogenous corticosterone oscillations during the circadian peak (see Materials and Methods). In the CRH-induced oscillations, there was a peak frequency of ∼1 pulse/h for all animals (Figure 3A–3B; mean peak frequency = 0.89 pulses/h; peak frequency range = 0.79–0.93 pulses/h). We then measured corticosterone levels in untreated control rats during the circadian peak, when endogenous CRH is maximal [34],[35] and corticosterone is pulsatile (Figure 1B). Corticosterone oscillations were observed in all animals (Figure 3C), and frequency analysis of the data (Figure 3D) revealed no significant difference between these endogenous oscillations and the oscillations induced by constant CRH infusion (*p*>0.57) (Figure 3E).

The consistency in oscillation frequency between different animals infused with constant CRH suggests that the oscillations are regulated by a generic mechanism at a sub-hypothalamic level. Furthermore, the maintenance of this dominant frequency component throughout the period of CRH infusion (Figure 3F) suggests that the underlying oscillator is deterministic—as opposed to stochastic—in agreement with our modelling hypothesis [33].

**Figure 4:**
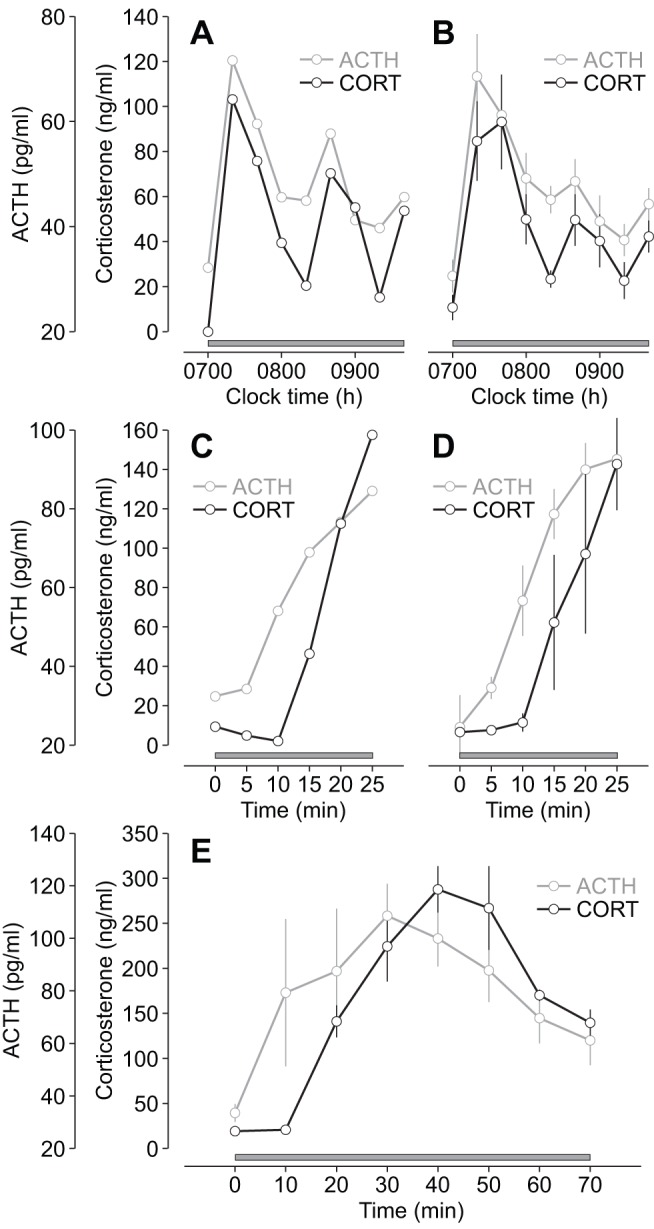
ACTH and glucocorticoid response to constant CRH infusion. (A–B) Individual (A) and mean (B) ACTH and corticosterone (CORT) oscillations in response to constant CRH infusion (0.5 µg/h; *n* = 6). (C–D) Individual (C) and mean (D) time course of the ACTH and corticosterone (CORT) response to constant CRH infusion (0.5 µg/h) during the initial activation phase (0–25 min) of the oscillation (*n* = 4). There was a significant overall effect of the CRH infusion on both ACTH and corticosterone (ACTH, *p*<0.0001; corticosterone, *p*<0.005; one-way ANOVA). ACTH was significantly different from basal (time zero) by 10 min (*p*<0.005), whereas corticosterone was not significantly different from basal (time zero) until 20 min (*p*<0.05). (E) Phase-shifted ACTH and corticosterone (CORT) response to constant CRH infusion (0.5 µg/h) over the duration of the first pulse (*n* = 3–7 per time point). Grey bar indicates the period of infusion (starting at 0700 h); error bars represent mean ± standard error of the mean (SEM).

Numerical simulations of the model indicate that glucocorticoid oscillations induced by constant CRH stimulation are driven by oscillations in ACTH (Figure 1C–1D). Ultradian ACTH oscillations have been observed in the rat [18], and have been shown to be a critical factor in regulating pulsatile glucocorticoid secretion from the adrenal cortex [38]. Moreover, coordinated ACTH and glucocorticoid oscillations have been observed in man [39]. To confirm that the constant CRH infusion generates oscillations in both hormones, we infused CRH (0.5 µg/h) from 0700–0940 h and measured circulating levels of both ACTH and corticosterone in samples collected at 20-min intervals throughout the infusion (see Materials and Methods). In agreement with the modelling predictions, CRH induced ACTH and corticosterone oscillations that persisted throughout the infusion period (Figure 4A–4B).

A key feature of the oscillation predicted by numerical simulations is a small phase shift between the two hormones—ACTH oscillations preceding glucocorticoid oscillations (Figure 1C–1D). This small phase shift could not be detected in the CRH-induced ACTH and corticosterone oscillations (Figure 4A–4B) because of the sampling frequency (20-min inter-sample interval). Therefore, we measured both ACTH and corticosterone at a higher sampling frequency (5-min inter-sample interval) over the first 25 min of the CRH infusion (0.5 µg/h), covering the initial activation phase of the first pulse. Both hormones rose rapidly in response to CRH (ACTH, *p*<0.0001; corticosterone, *p*<0.005), with the ACTH increase preceding a delayed rise in corticosterone (Figure 4C–4D). Specifically, ACTH was significantly different from basal (time zero) by 10 min (*p*<0.005), whereas corticosterone was not significantly different from basal (time zero) until 20 min (*p*<0.05). This phase shift between ACTH and corticosterone presumably reflects the time taken for de novo biosynthesis and release of corticosterone from the adrenal cortex [40].

We then checked that this phase relationship is maintained over the full pituitary-adrenal oscillation. Since collection of large volumes of plasma (required for sensitive ACTH assay) can activate a stress response [41], this precludes high-frequency blood sampling (for ACTH measurement) for prolonged periods in the rat. We therefore used an alternative experimental approach in which animals were killed by decapitation at 10-min intervals throughout the first 70 min of the CRH infusion (0.5 µg/h); ACTH and corticosterone levels were measured in plasma obtained from trunk blood (see Materials and Methods). As observed in the case of high-frequency sampling (Figure 4C–4D), the CRH-induced increase in corticosterone was delayed relative to the increase in ACTH, and this phase shift was maintained over the duration of the pulse (Figure 4E).

These results challenge the long-standing view that glucocorticoid oscillations are a consequence of pulsatile CRH secretion from a neuronal “pulse generator” within the hypothalamus [26],[27]. Our approach was based on the premise that feedback is a key regulatory feature of biological oscillators [42], and on our numerical results [33], suggesting that a systems-level dynamic balance between positive feedforward and negative feedback—CRH stimulation against rapid glucocorticoid inhibition of ACTH secretion—provides a mechanism for generating ultradian oscillations in ACTH and glucocorticoid hormone secretion. Testing this modelling prediction in vivo, our results show that constant CRH stimulation of the anterior pituitary is sufficient to generate ACTH and glucocorticoid oscillations at a physiological ultradian frequency, providing good evidence for an oscillating mechanism outside the central nervous system.

The hormone oscillations generated by this system are not simply a dynamic epiphenomenon of the pituitary-adrenal interaction, but have significant biological impact. Glucocorticoid oscillations are essential for optimal transcriptional regulation [20],[21], and are also likely to be important for more rapid non-transcriptional mechanisms of steroid action in the brain [43] that can alter behavioural function within minutes [44],[45]. Exposure of tissues to abnormal glucocorticoid levels due to prolonged stress [46] and raised CRH [47],[48], or due to the loss of ultradian pulsatility that has been detected in ageing animals [49], will modify glucocorticoid signalling, and could be an important factor for both stress- and age-related disease.

## Materials and Methods

### Subjects

All experiments were conducted on adult male Sprague-Dawley rats (Harlan Laboratories, Inc.) weighing ∼250 g at the time of surgery. Animals were housed in groups of four per cage and were given at least 1 wk to acclimatize to the housing facility prior to surgery. Rats were maintained under standard environmental conditions (21±1°C) under a 14-h light, 10-h dark schedule (lights on at 0500 h). Food and water were freely available throughout the experiments. All animal procedures were conducted in accordance with Home Office guidelines and the UK Animals (Scientific Procedures) Act, 1986.

### Surgical Procedures

Animals were anaesthetized with a combination of Hypnorm (0.32 mg/kg fentanyl citrate and 10 mg/kg fluanisone, IM; Janssen Pharmaceuticals) and diazepam (2.6 mg/kg, IP; Phoenix Pharmaceuticals). Two silastic-tipped (Merck) polythene cannulae (Portex) were pre-filled with pyrogen-free heparinized (10 IU/ml) isotonic saline. The right jugular vein was exposed and both cannulae inserted into the vessel until they lay close to the entrance to the right atrium. This permits simultaneous intravenous blood sampling (via the sampling cannula) and substance infusion (via the infusion cannula). The free ends of the cannulae were exteriorized through a scalp incision and tunnelled through a protective spring that was anchored to the parietal bones using two stainless steel screws and self-curing dental acrylic. Following recovery, animals were individually housed in a room containing an automated blood-sampling system. The end of the protective spring was attached to a two-channel swivel (Instech Laboratories, Inc.), which rotates through 360° in the horizontal plane and 180° in the vertical plane, providing the animals with maximal freedom of movement. Animals were given a 5-d recovery period prior to experiments. Throughout this time, both cannulae were flushed daily with heparinized saline to maintain patency.

### Drug Infusions

Rats received a constant intravenous infusion of either 0.9% saline solution (vehicle control animals) or synthetic human/rat CRH (University of Bristol Peptide Synthesis Service) dissolved in 0.9% saline solution. In all experiments, drugs were infused through the infusion cannula at a volume infusion rate of 0.5 ml/h using an automated infusion pump (PHD ULTRA syringe pump; Harvard Apparatus, Ltd.).

### Experimental Design

In the experiments measuring basal corticosterone levels over 24 h, corticosterone levels in response to saline or CRH infusion, or basal corticosterone oscillations during the circadian peak, blood samples were collected from the sampling cannula using an automated blood-sampling system [50]. In the experiment measuring basal corticosterone levels over 24 h (Figure 1B), blood samples were collected every 10 min from 1400–1350 h. In the experiments measuring corticosterone levels in response to saline or CRH infusion (Figures 2A–2H, 2J, and 3A), rats were constantly infused with saline or CRH (0.5–2.5 µg/h) from 0700–1300 h; blood samples were collected every 5 min from 0600–1300 h. In the experiments measuring basal corticosterone oscillations during the circadian peak (Figure 3C), blood samples were collected every 5 min from 1530–2100 h. At the end of each experiment, the plasma fraction was separated by centrifugation (15 min, 3,120 *g*, 4°C) and stored at −20°C until processed for corticosterone measurement.

In the experiments measuring ACTH and corticosterone oscillations in response to CRH infusion (Figure 4A–4B), rats received a constant CRH infusion (0.5 µg/h) from 0700–0940 h and blood samples were collected manually from the sampling cannula every 20 min throughout the infusion. In the experiments measuring ACTH and corticosterone levels in response to CRH during the initial activation phase (Figure 4C–4D), rats received a constant CRH infusion (0.5 µg/h) from 0700–0725 h and blood samples were collected manually from the sampling cannula every 5 min throughout the infusion. Blood samples from both experiments were immediately mixed with EDTA (10 µl, 0.5 M, pH 7.4) and placed on crushed ice. The plasma fraction was separated by centrifugation (15 min, 3,120 *g*, 4°C) within 20 min of sample collection and stored at −20°C until processed for ACTH and corticosterone measurement.

In the experiment measuring ACTH and corticosterone levels in response to CRH infusion over the first pulse (Figure 4E), rats received a constant CRH infusion (0.5 µg/h) from 0700–0810 h. At 10-min intervals throughout the CRH infusion, rats were killed by decapitation following an overdose of 0.3 ml sodium pentobarbital (Euthatal, 200 mg/ml; Merial). Trunk blood was collected and immediately mixed with EDTA (50 µl, 0.5 M, pH 7.4) and placed on crushed ice. The plasma fraction was separated by centrifugation (15 min, 3,120 *g*, 4°C) within 20 min of sample collection and stored at −20°C until processed for ACTH and corticosterone measurement.

### Corticosterone RIA

Corticosterone was measured in plasma by radioimmunoassay (RIA) [51]. Samples were diluted in a citrate buffer (pH 3.0) to denature the binding globulin. Antisera was supplied by G Makara (Institute of Experimental Medicine, Budapest, Hungary), and [125I] corticosterone was purchased from Izotop (Institute of Isotopes Co. Ltd., Budapest, Hungary). The intra- and inter-assay coefficients of variation of the corticosterone RIA were 14.1% and 15.3%, respectively.

### ACTH IRMA

ACTH was measured in plasma by immunoradiometric assay (IRMA; DiaSorin Ltd.), in accordance with the manufacturer's protocol. The intra- and inter-assay coefficients of variation of the ACTH IRMA were 2.8% and 6.4%, respectively.

### Data Analysis

Overall corticosterone responses to saline or CRH infusion were assessed by AUC. Characterization of oscillatory corticosterone responses induced by CRH infusion, and of endogenous corticosterone oscillations recorded during the circadian peak, was performed using spectral methods. Missing data points were linearly interpolated and data were detrended using the Smoothness Priors Approach (SPA) [52] with the smoothing parameter set at λ = 30. This parameter value was chosen so as to remove long term changes in the mean (i.e., low-frequency fluctuations), while keeping the higher-frequency ultradian fluctuations that were the focus in this study. To define the frequency of the oscillatory corticosterone data, we computed the power spectrum of the detrended data using the Discrete Fourier Transform (DFT) applied to a time window corresponding to the period of CRH infusion (0700–1300 h), or to the period of sampling (1530–2100 h) for the basal corticosterone oscillations recorded during the circadian peak. The peak frequency was then taken as the frequency value corresponding to the maximum spectral power of the DFT, which was calculated using a quadratic interpolation. Spectrograms were computed using the Short-Time Fourier Transform (STFT).

### Statistical Analysis

Statistical significance level was set at *p*<0.05. Saline-infused corticosterone responses and untreated corticosterone profiles were compared using the non-parametric Mann-Whitney U test. The overall effect of CRH treatment on AUC was assessed using the non-parametric Kruskal-Wallis ANOVA on ranks test, and post hoc multiple comparisons were performed using the Mann-Whitney U Test with the statistical significance level adjusted using the Bonferroni correction. Peak frequencies obtained using the DFT, for the groups with CRH-induced corticosterone oscillations and endogenous corticosterone oscillations during the circadian peak, were compared using the Mann-Whitney U test. The ACTH and corticosterone response to constant CRH infusion was analyzed using one-way ANOVA, followed by Fisher Least Significant Difference (LSD) post hoc test.

## Abbreviations

ACTH: adrenocorticotrophic hormone
AUC: area under the curve
CORT: glucocorticoids/corticosterone
CRH: corticotrophin-releasing hormone
HPA: hypothalamic-pituitary-adrenal
